# Conformation-dependent binding of a Tetrastatin peptide to α_v_β_3_ integrin decreases melanoma progression through FAK/PI_3_K/Akt pathway inhibition

**DOI:** 10.1038/s41598-018-28003-x

**Published:** 2018-06-29

**Authors:** Eléonore Lambert, Eloïse Fuselier, Laurent Ramont, Bertrand Brassart, Sylvain Dukic, Jean-Baptiste Oudart, Aurélie Dupont-Deshorgue, Christèle Sellier, Carine Machado, Manuel Dauchez, Jean-Claude Monboisse, François-Xavier Maquart, Stéphanie Baud, Sylvie Brassart-Pasco

**Affiliations:** 1 UMR CNRS/URCA 7369, Matrice Extracellulaire et Dynamique Cellulaire (MEDyC), Université de Reims Champagne Ardenne (URCA), Reims, F-51100 France; 2 CHU de Reims, Laboratoire Central de Biochimie, Reims, F-51092 France; 3 CNRS UMR 7312, Institut de Chimie Moléculaire de Reims, Université de Reims Champagne Ardenne (URCA), Reims, F-51100 France; 4 Plateau de Modélisation Moléculaire Multi-échelle, Université de Reims Champagne Ardenne (URCA), Reims, F-51687 France; 5 Present Address: Laboratoire de Recherche sur les Nanosciences (LRN), EA4682, Université de Reims Champagne-Ardenne, Reims, F-51685 Reims, France

## Abstract

Tetrastatin, a 230 amino acid sequence from collagen IV, was previously demonstrated to inhibit melanoma progression. In the present paper, we identified the minimal active sequence (QKISRCQVCVKYS: QS-13) that reproduced the anti-tumor effects of whole Tetrastatin *in vivo* and *in vitro* on melanoma cell proliferation, migration and invasion. We demonstrated that QS-13 binds to SK-MEL-28 melanoma cells through the α_v_β_3_ integrin using blocking antibody and β3 integrin subunit siRNAs strategies. Relevant QS-13 conformations were extracted from molecular dynamics simulations and their interactions with α_V_β_3_ integrin were analyzed by docking experiments to determine the binding areas and the QS-13 amino acids crucial for the binding. The *in silico* results were confirmed by *in vitro* experiments. Indeed, QS-13 binding to SK-MEL-28 was dependent on the presence of a disulfide-bound as shown by mass spectroscopy and the binding site on α_V_β_3_ was located in close vicinity to the RGD binding site. QS-13 binding inhibits the FAK/PI_3_K/Akt pathway, a transduction pathway that is largely involved in tumor cell proliferation and migration. Taken together, our results demonstrate that the QS-13 peptide binds α_v_β_3_ integrin in a conformation-dependent manner and is a potent antitumor agent that could target cancer cells through α_V_β_3_.

## Introduction

The extracellular matrix (ECM) is a complex structure that is composed of many proteins, proteoglycans and hyaluronic acid. Basement membranes, which are specialized ECMs, are composed of type IV collagen in association with minor collagens, laminins, nidogens and perlecan^1^. Type IV collagen is composed of three α chains, out of six possible chains (α1(IV)-α6(IV))^2^. Type IV collagen contains a 7S N-terminal domain, an interrupted triple helical domain and a globular C-terminal non-collagenous (NC1) domain^3^.

Tumor invasion and metastasis require proteolytic degradation of the ECM involving various proteolytic cascades, such as matrix metalloproteinases (MMP) and the plasminogen/plasmin system. Tumor progression is controlled by the tumor microenvironment, including several intact ECM macromolecules and/or fragments called matrikines^4^. Among them, the NC1 domains of several collagen chains have been shown to inhibit angiogenesis and tumor growth^5–10^ *via* integrin binding and through the FAK/PI_3_K/Akt pathway^10–16^. Matrikine binding to the receptor and biological activity appear to be conformation dependent^17,18^.

The NC1 α4(IV) domain, named Tetrastatin, was shown to exert potent anti-tumor activity both *in vitro* and *in vivo* in a human melanoma model by decreasing the proliferative and invasive properties of melanoma cells through an α_v_β_3_ integrin-dependant mechanism. We also demonstrated that the last fifty amino-acids of Tetrastatin (AA 180–229, named CS-50) were able to reproduce its inhibitory effects on cell proliferation and invasion *in vitro*^19^. The aim of the present study was to identify a shorter fragment that is able to reproduce the Tetrastatin anti-tumor activity and to determine the molecular mechanisms involved.

## Results

### Identification of a 13-amino-acid peptide from Tetrastatin that inhibits ***in vivo*** tumor growth in a mouse melanoma model

**Figure 1:**
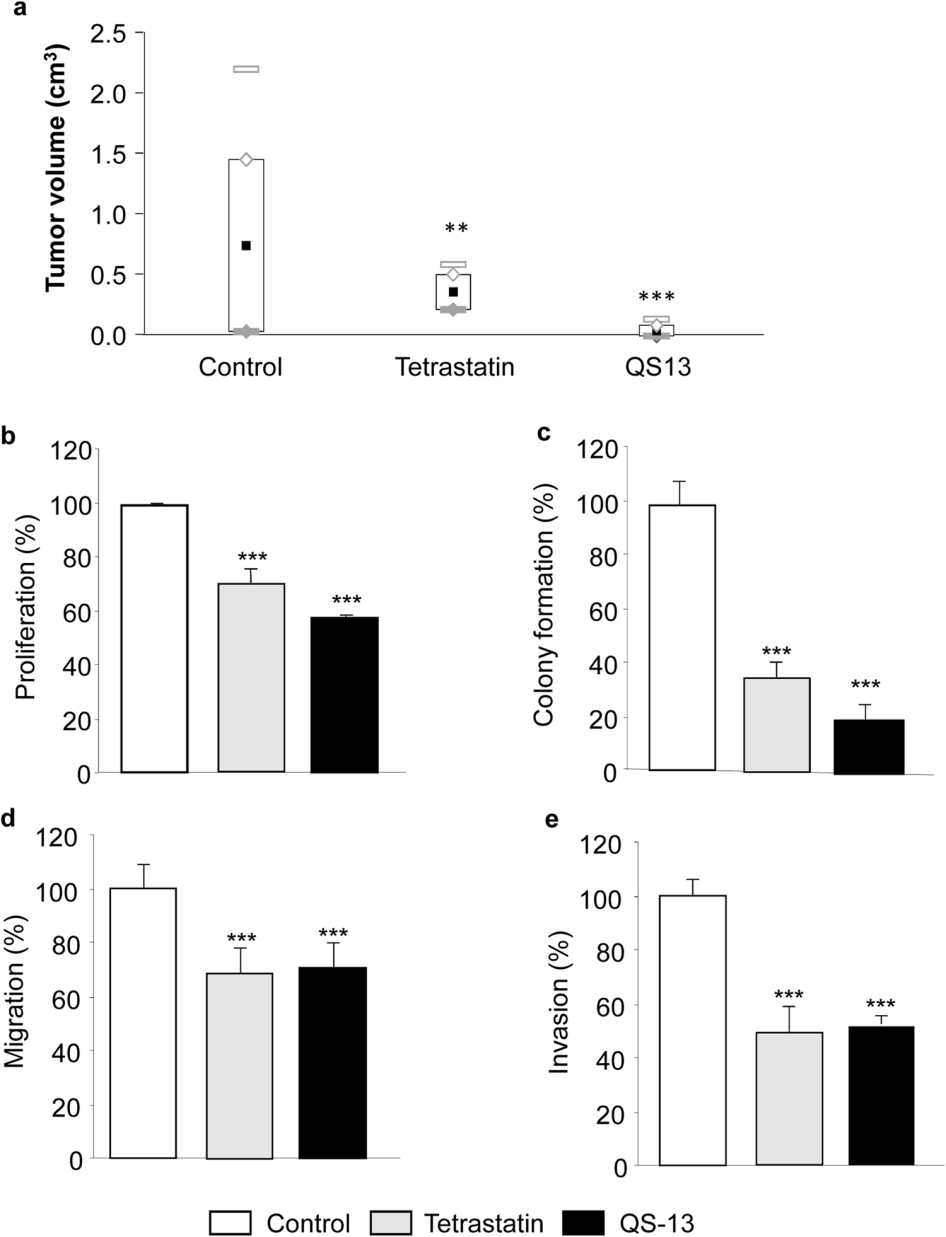
QS-13 peptide inhibits *in vivo* tumor growth, *in vitro* SK-MEL-28 melanoma cell proliferation, colony formation, migration and invasion. Tumor growth was measured at day 20 in a mouse melanoma model (**a**). Cell proliferation was measured after 72 h of incubation (**b**). Colony formation in soft agar was measured after 10 days of incubation (**c**). Cell migration in scratch wound assay was measured after 48 h of incubation. (**d**) Cell invasion through Matrigel-coated membranes was measured after 48 h of incubation (**e**) **p < 0.01, ***p < 0.001.

B16-F1 cells were subcutaneously injected into the left side of C57Bl6 mice and the tumor volume was measured at days 10, 15 and 20. Tetrastatin and CS-50 treatments induced a decrease in tumor volume of 51 and 52%, respectively, at day 20 versus control. The N-terminal 13-amino acid fragment from Tetrastatin (AA 217–229), named QS-13, inhibited tumor growth by 95% (Fig. 1a).

### QS-13 inhibits *in vitro* melanoma cell proliferation, migration and invasion

SK-MEL-28 cell proliferation was measured using WST-1 as the reagent. After 72 h of incubation with peptides, cell proliferation was inhibited by 30% with Tetrastatin and 26% with the CS-50 peptide. QS-13 inhibited cell proliferation by 42% (Fig. 1b). The different peptides were also tested on SK-MEL-28 colony formation in soft agar. Tetrastatin and CS-50 inhibited cell growth by 64 and 62%, respectively, whereas the inhibitory effect was 80% with the QS-13 peptide (Fig. 1c). In an artificial wound assay, Tetrastatin, CS-50 and QS-13 inhibited cell migration by 27%, 30% and 30%, respectively (Fig. 1d). In modified Boyden chambers with Matrigel-coated membranes, Tetrastatin, CS-50 and QS-13 inhibited SK-MEL-28 cell invasion by 52%, 44% and 49.5%, respectively (Fig. 1e). Our results demonstrate that the QS-13 peptide reproduces the Tetrastatin inhibitory effects *in vitro*.

### QS-13 peptide binds to SK-MEL-28 through the α_v_β_3_ integrin

**Figure 2:**
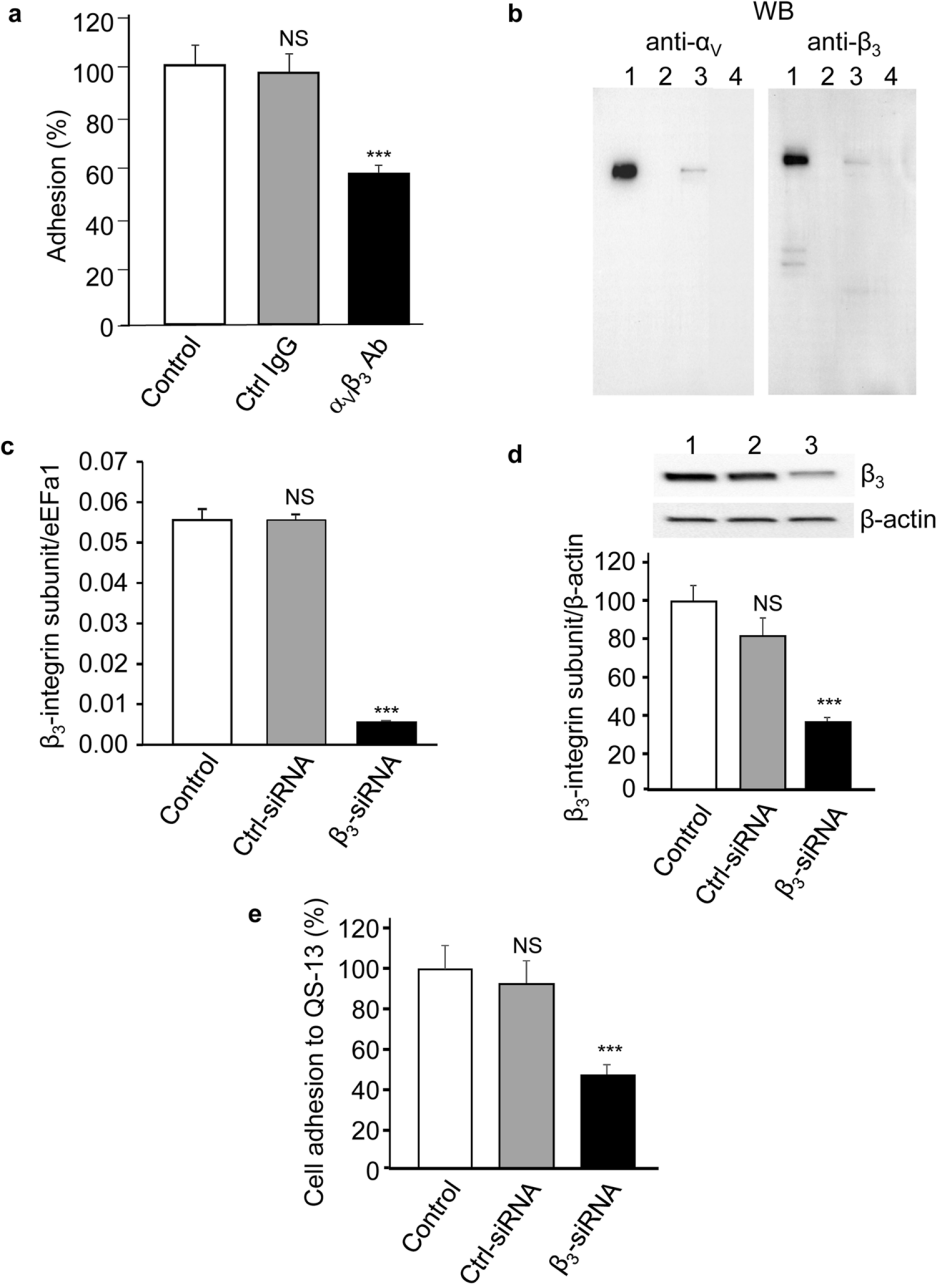
QS-13 peptide binds to SK-MEL-28 through α_v_β_3_ integrin. Cells were pre-incubated for 30 min with an anti-α_v_β_3_ blocking antibody or an irrelevant IgG (10 µg/mL) before seeding on QS-13 peptide and adhesion was assessed (**a**). Cell extracts were submitted to affinity chromatography on a QS-13 peptide-bounded column and analyzed by western blot. Total cell extract (line 1), fraction eluted with 0.15 M (line 2), 0.6 M (line 3), 1 M NaCl (line 4) (**b**). Cells were transfected with β_3_ subunit siRNA or control siRNA. Gene expression was measured by RT-qPCR 24 h after transfection (**c**). Protein expression was assessed by western-blot 48 h after transfection. Lane 1: control; lane 2: control siRNA; lane 3: β_3_ subunit siRNA (**d**). Cells were seeded on QS-13 peptide 48 h after transfection and adhesion was measured (**e**) ***p < 0.001.

To determine whether QS-13 binds to the α_v_β_3_ integrin on melanoma cells, we measured SK-MEL-28 cell adhesion on QS-13 peptides in the presence or absence of an anti-α_v_β_3_ integrin blocking antibody (10 µg/mL). The blocking anti-α_v_β_3_ antibody significantly inhibited (40%) cell adhesion on QS-13, whereas an irrelevant IgG had no effect (Fig. 2a).

Moreover, SK-MEL-28 extracts were analysed by QS-13 peptide affinity chromatography. Proteins bound to the affinity column were eluted with increasing concentrations of NaCl (0.15, 0.6 and 1.0 M). Eluted samples were submitted to sodium dodecyl sulfate (SDS)-poly-acrylamide gel electrophoresis (PAGE) and analysed by western blot. The 0.6 M eluted samples revealed bands that matched the recombinant α_v_ and β_3_ integrin subunits (Fig. 2b).

To confirm that the α_v_β_3_ integrin acts as a receptor for QS-13 on SK-MEL-28 cells, β_3_ integrin expression was decreased by specific siRNA transfection. The β_3_ integrin mRNA and protein levels were evaluated by RT-qPCR and western blot, respectively. β_3_ integrin siRNA transfection decreased β_3_ integrin mRNA expression by 91% (Fig. 2c) and protein expression by 65% (Fig. 2d, supplementary Fig. S1). Control siRNAs did not significantly modify β_3_ subunit expression. Transfected cells were seeded on the QS-13 peptide. Control siRNAs did not significantly modify cell adhesion. By contrast, β_3_ subunit siRNAs decreased cell adhesion by 50% (Fig. 2d). Taken together, our results confirm that the α_v_β_3_ integrin is a receptor of QS-13 on SK-MEL-28 cells.

### QS-13 peptide presents a disulfide bond in solution

**Figure 3:**
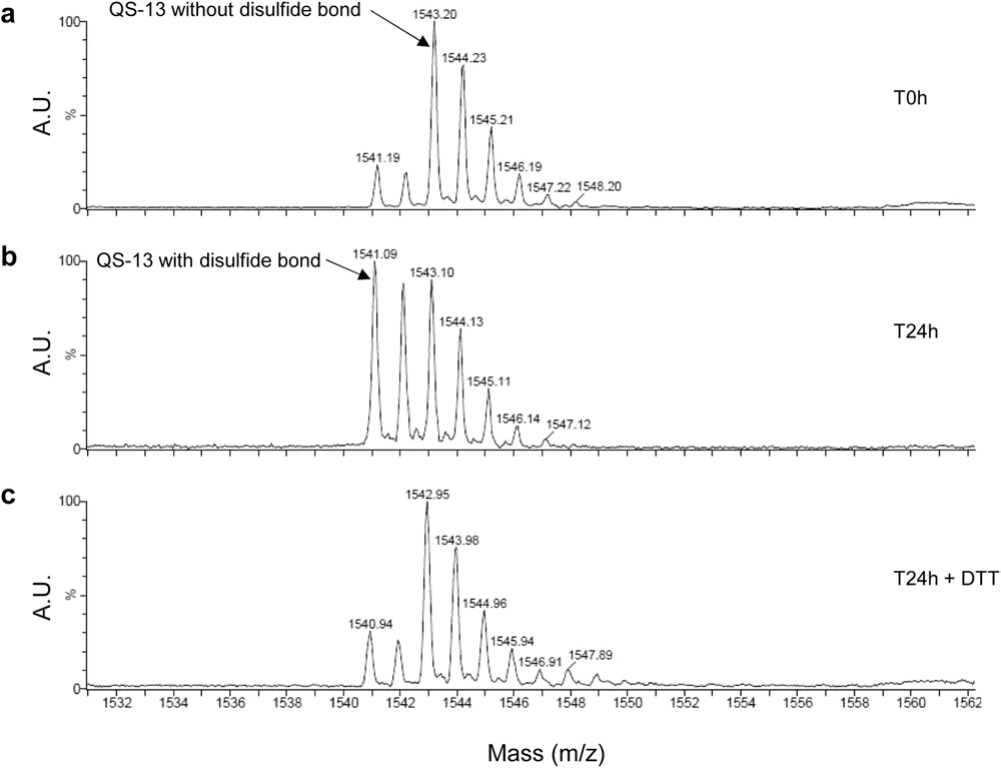
QS-13 peptide forms intra chain disulfide bond in solution. MALDI-ToF MS analysis of the QS-13 peptide at t 0 h (no disulfide bond) (**a**), t 24 h (presence of disulfide bond) (**b**), t 24 h + dithiothreitol (DTT) (no disulfide bond) (**c**).

MALDI-ToF MS analyses demonstrated that the QS-13 peptide (m/z 1543) (Fig. 3a) formed an intra-chain disulfide-bond between ^222^C and ^225^C of Tetrastatin in solution over time (Fig. 3b). The disulfide-bond was reduced by dithiothreïtol (Fig. 3c).

### Molecular dynamics (MD) simulation of QS-13

**Figure 4:**
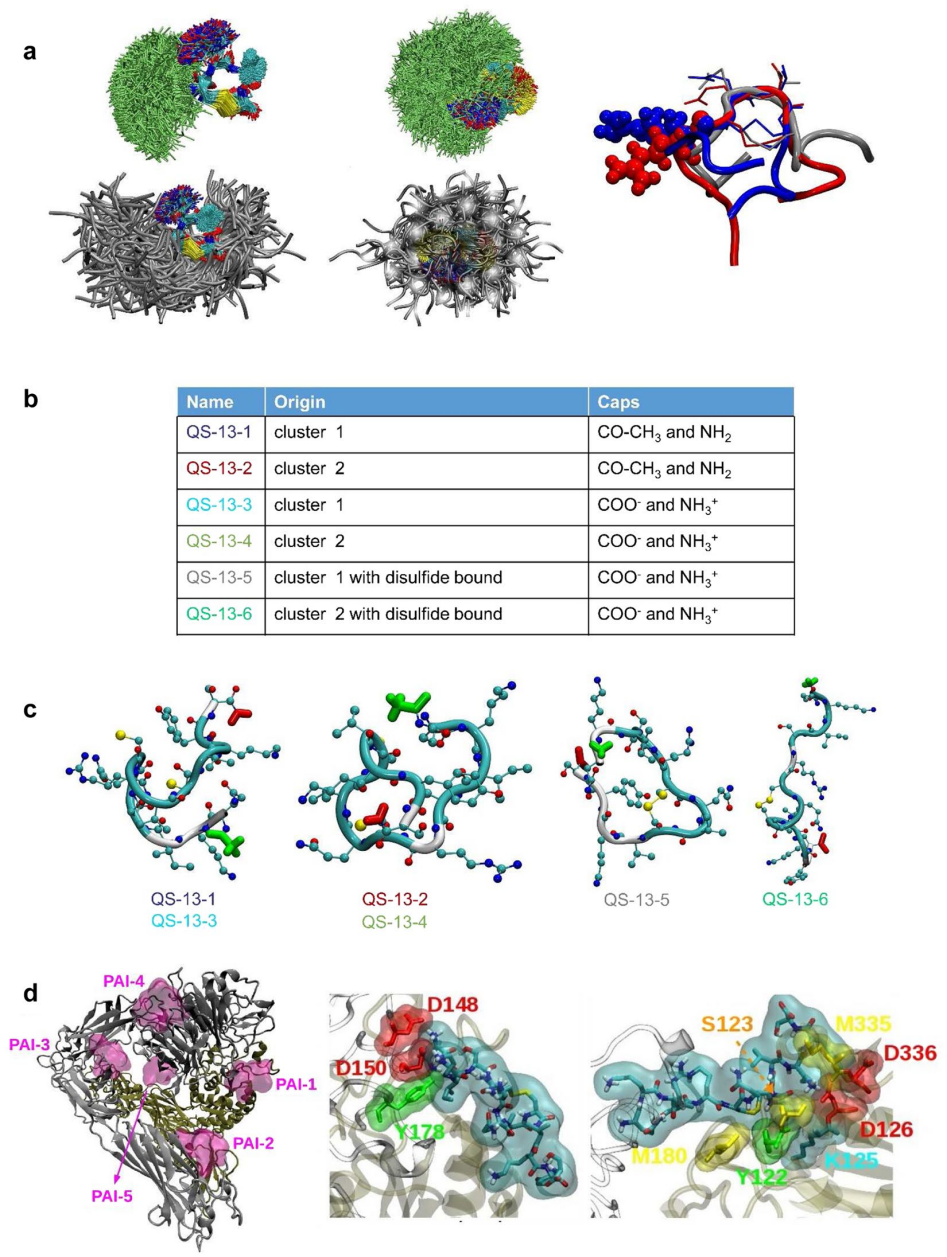
Molecular Dynamics simulation and docking of the QS-13 peptide. (**a**) The snapshots extracted from MD simulations of the QS-13-db (left panel) and QS-13 (central panel) peptides were superimposed using the α carbons of the CQVC central sequence. A first type of display (upper panel) is obtained with the licorice representation picturing the CQVC central residues (atom color coded) and the Arginine residue (in light green). The second type of display (lower panel) is obtained using the licorice representation for the CQVC central residues (atom color coded) and the new cartoon representation for the backbone (in silver). Finally, superimposition of the first cluster of the disulfide bonded peptides was performed (right panel). The structures of the AS-13-db (in red), QS-13-R5A-db (in silver) and QS-13-db (in blue) were superimposed using the α carbons of the CQVC central sequence. The backbone is displayed using the new cartoon representation, the CQVC sequence using the licorice representation and the Arginine residues using the Van der Waals representation. (**b**) Specification of the QS-13 peptides characteritics related to the origin of the cluster (family of conformation) and the nature of the termini. (**c**) Representative conformation of QS-13 peptides. N-termini are displayed in green licorice and C-termini in red licorice. The backbone of the peptides is colored according to its local secondary structure (cyan for turn and white for coil) and the sulfur atoms are shown as yellow spheres. (**d**) The results of the QS-13-3 docking experiment highlight the existence of 5 Preferred Area of Interaction (PAI) (magenta surfaces). These PAI are also evidenced through the docking experiments performed with the other peptides. The protein is represented using the New Cartoon scheme and colored according to the nature of the chain (grey for α_V_ and brown for β_3_) (left panel). Identification of contacts between QS-13-6 and the PAI shared with RGD peptides: contacts made with the α_V_ subunit (central panel) and contacts made with the β3 subunit (right panel).

The first objective of the MD simulation was to explore and characterize the intrinsic structural behaviour of the investigated peptides, specifically the role of the experimentally evidenced disulfide bond and potential key residues. The second objective was to select relevant QS-13 conformations that can be used for docking experiments on integrin α_v_β_3_. From the different 200 ns NPT simulations performed on a set of 10 different peptides (Supplementary Table S1), clustering analyses allowed us to identify the different types of conformations that occurred along the trajectory. One striking observation was that the presence of the disulfide bond between the ^222^C and ^225^C was directly responsible for the occurrence of a lower number of clusters (see Supplementary Table S2). Indeed, for the four peptides presenting a disulfide bond, there was at least one division by two of the number of clusters compared to the peptide without the disulfide bond. In addition, the observation and superposition of the MD trajectory showed that the structure of the **C**QV**C** portion was almost frozen in the same conformation (Fig. 4a, left panel) for these 4 peptides and that the Q and V middle residues never explored a coil conformation, favouring a bend or a turn local structure. In addition, transitioning from the disulfide bond structure to the structure without the disulfide bond, the increase in the number of clusters was accompanied by a decrease in the percentage of structures found in the five first clusters.

Comparing the structures with (Fig. 4a, left panel) and without (Fig. 4a, central panel) the disulfide bond, it is quite understandable that since the peptides without the disulfide bond “explore” a larger configuration space, they are characterized by a larger number of clusters. Another interesting feature is illustrated in the right panel of Fig. 4a: not only is the structure of the **C**QV**C** portion almost frozen for a given peptide, but by aligning the representative structures (i.e., the first cluster) of the four peptides containing a disulfide bond, we showed that they fit very well and that they almost conserved the same orientation and exposure of the QV and R side chains towards the outside of the bond.

When we analysed the global content of the secondary structures, the major observation was an increase of the coil structure when the disulfide bond was present. An increase of the β-bridge or β-sheet structure were found when cysteine or arginine residues were mutated into alanine residues.

The different clusters studied below are listed in Fig. 4b. Figure 4c presents the secondary structures of QS-13 first and second clusters of the QS-13 peptide that were used in the docking experiments. From these representations, we observed that the disulfide bond locked CQVC in a conformation that exposed the glutamine side chain. The side chain exposure was explored systematically for all of the MD simulations (Supplementary Fig. S2). Two factors strongly modified the general profile of the solvent accessible surface (SAS) of the peptide: the loss of the disulfide bond and modification of glutamine, arginine or cysteine residues, especially, when one of the cysteine residues was mutated into alanine.

### Docking experiments of QS-13 on α_V_β_3_

For each QS-13 peptide (listed in Fig. 4b), we collected 27000 (180 × 150) structures from the docking experiments. To overcome the bias introduced by the forced exploration of empty or unfavourable interaction areas, we kept the first 180 lowest energy structures and studied them in detail. These structures were then spatially regrouped in clusters to allow identification of the most probable interacting surface protein areas. For each QS-13 peptide, we identified the preferred areas of interaction (PAI) with the integrin; at the most, five were identified. In the left panel of Fig. 4d, the five PAIs found with the QS-13–3 peptide (also representative of the results obtained with the other peptides) are shown.

Most often for 4 QS-13 peptides out of the 6 investigated through docking, as shown in Fig. 4b, PAI-5 was the area that had the highest frequency (see Table S3). From the energetic point of view, the negative binding energies accounted for the favourable interactions between the QS-13 peptide and this area of α_v_β_3_. On average, PAI-3 was the least populated area. It was not explored by the QS-13-2 peptide, and in 3 of the 5 experiments, the lowest binding energy did not traduce any favourable interaction since it was close to zero. Other than PAI-3, which could be removed from the potential interacting areas, it seemed difficult to remove the remaining PAI. Looking at the energy, 2 out of the 6 peptides seemed to be very interesting, QS-13-5 and QS-13-6, since they had at least 4 of the lowest binding energies below the threshold of −3.00 kcal/mol. New docking experiments were performed with bonded QS-13, positioning the centres of the grid search on the centre of mass of each PAI. Following this protocol, we were able to identify the residues of the integrin that interacted directly the QS-13 peptides (central and right panel of Fig. 4d). It was interesting to observe that among the residues that made contact with the QS-13 peptide, the aspartic acid residue was one of the most common, followed by glutamic acid, tyrosine and serine. Focusing the contact investigation on the best binding energy structure obtained in the PAI-1 region, we again highlight the fact that the contacts made by QS-13 mostly involved these four types of residues in the protein. Finally, when we compare our docking results with 1L5G PDB structure^20^, we observe that the position of PAI-1 is colocalized with the region of interaction between the RGD peptide and the integrin.

### RGDS peptide inhibits QS-13 binding to SK-MEL-28 cells

**Figure 5:**
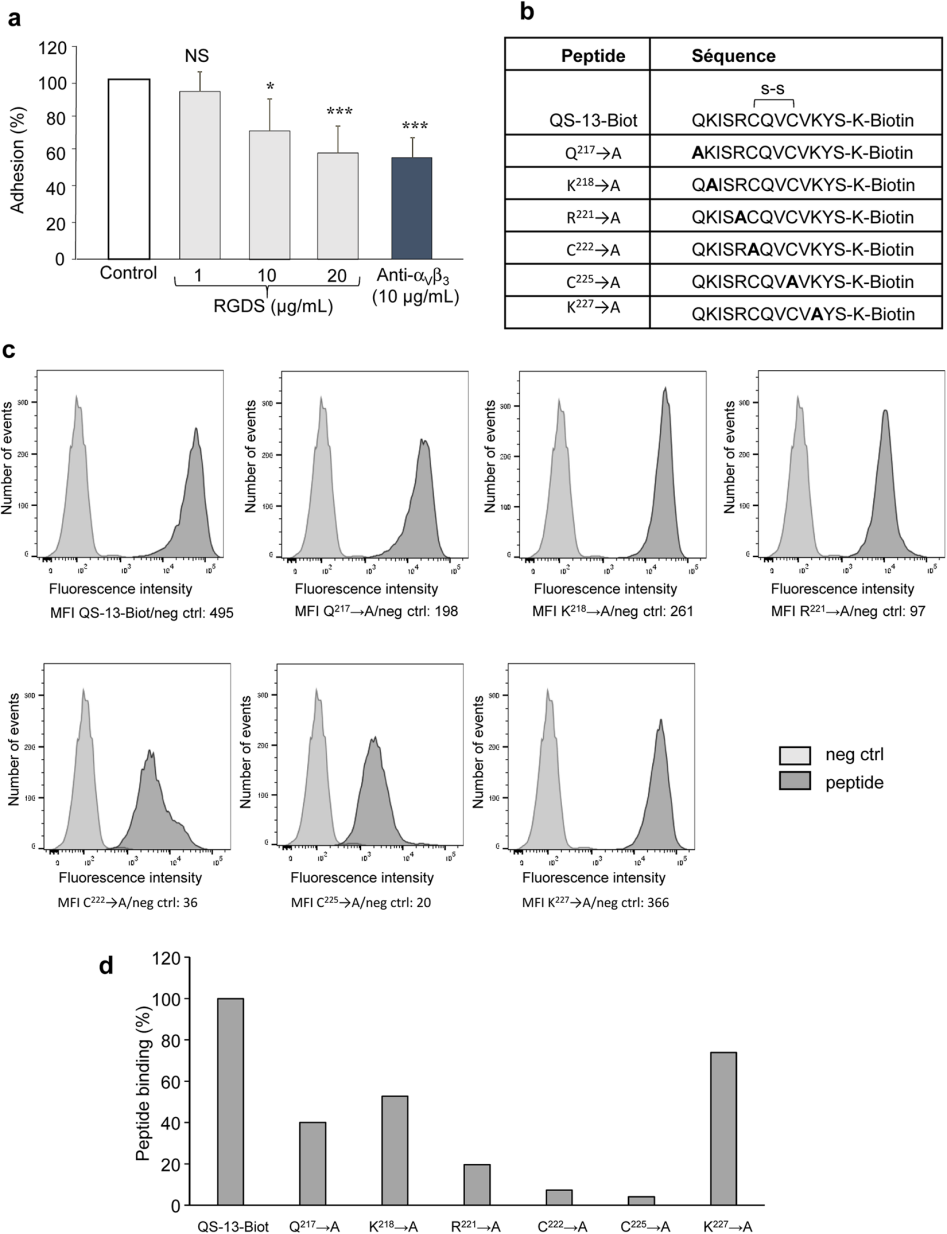
Characterization of the QS-13 residues crucial for α_v_β_3_ integrin interaction. SK-MEL-28 melanoma cells were pre-incubated 30 min in the presence or absence of the RGDS peptide (1 to 20 µg/mL) or anti-α_v_β_3_ blocking antibody (20 µg/mL) and adhesion was assessed. *p < 0.05, ***p < 0.01, ***p < 0.001 (**a**). SK-MEL-28 melanoma cells were incubated with different substituted peptides (**b**) tagged with biotin and with AF-488 streptavidin complex and analyzed by flow cytometry (**c**) and results are reported on a histogram (**d**).

Based on the docking experiments, we evidence that one of the five hypothetical QS-13-binding areas (PAI-1) overlaps the RGD binding site^20^. To test this hypothesis, SK-MEL-28 melanoma cells were preincubated with different concentrations of RGDS peptide (from 1 to 20 µg/mL) or with an anti-α_v_β_3_ integrin blocking antibody (10 µg/mL) and seeded on QS-13 coated 96-well plates. As shown in Fig. 5a, cell preincubation with RGDS inhibited cell adhesion to the QS-13 peptide similarly to the anti-α_v_β_3_ integrin blocking antibody. These results provide evidence that the QS-13 binding site may be the RGD binding site or a new site in close proximity to the RGD binding site on integrin α_v_β_3_ and suggest that the first theoretical area (PAI-1) identified by the docking experiments may represent the real binding site.

### ^222^C and ^225^C of Tetrastatin are crucial for QS-13 binding to SK-MEL-28 cells

Molecular dynamics simulation and docking experiments highlighted the importance of several QS-13 sequences in binding to the α_v_β_3_ integrin. Six substituted peptides labelled with biotin on their C-terminus were synthesized (Fig. 5b). Their binding to SK-MEL-28 cells was evaluated by flow cytometry (Fig. 5c). The different substitutions decreased peptide binding to α_v_β_3_ (Fig. 5d). Substitution of ^222^C and ^225^C completely abolished the adhesion and by the way, cell proliferation, migration and invasion (Supplementary Fig. S3). By mass spectroscopy, we showed that the QS-13 peptide forms an intrachain disulfide bond that appeared to be crucial for QS-13 binding to cell surface. The substitution of ^221^R completely abolished the adhesion. The ^221^R residue might also be crucial for the binding.

### QS-13 binding to αvβ3 inhibits FAK, PI3-kinase and Akt phosphorylation

**Figure 6:**
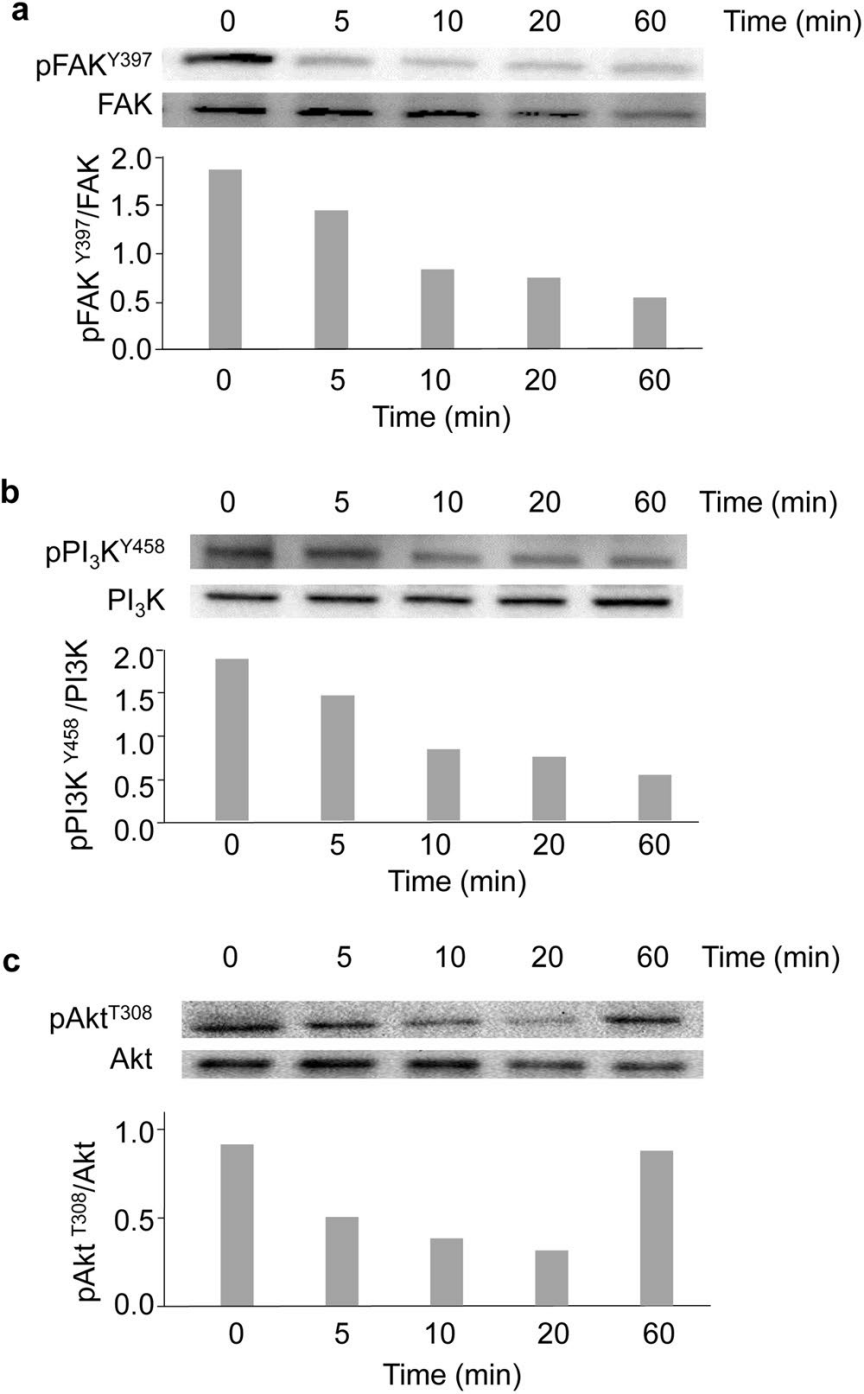
Kinetic analysis of FAK, PI_3_K p85 and Akt phosphorylation in SK-MEL-28 cells after incubation with QS-13. Western blot analysis of phosphorylated-FAK^Y397^ compared to total FAK (**a**), phosphorylated-PI_3_K p85^Y458^ compared to total PI_3_K p85 subunit (**b**), phosphorylated-Akt^T308^ compared to total Akt (**c**). Bands were quantified by densitometric analysis and phosphorylated proteins were reported to corresponding total proteins.

It is now well established that the FAK/PI_3_K/Akt pathway plays a critical role in the progression of melanoma. To analyse the effects of QS-13 on this transduction pathway, SK-MEL-28 melanoma cells were incubated with the QS-13 peptide for 5, 10, 20, 60 min. The expression of total proteins and their corresponding phosphorylated forms were evaluated by western blotting. The FAK^Y397^/total FAK ratio was decreased by 67% at 5 min and 76% at 10 min (Fig. 6a). The PI_3_K p85^Y458^/total PI_3_K p85 ratio decreased gradually from 33% at 5 min to 72% at 60 min (Fig. 6b). The pAkt^T308^/total Akt ratio decreased gradually from 46% after 5 min of incubation to 66% after 20 min (Fig. 6c). Taken together, our results demonstrated that the FAK/PI_3_K/Akt pathway is impacted by the QS-13 peptide (Supplementary Fig. S4).

## Discussion

Although the basement membrane provides structural support for epithelial and endothelial cells, many studies have demonstrated that it also acts as a potential regulator of cell behaviour. Type IV collagen is a major component of basement membranes and plays a pivotal role in the regulation of cell proliferation, adhesion and migration, either through its triple helical domain^21,22^ or through the NC1 domain of its constitutive α(IV) chains^4,23^. We demonstrated that the NC1 α4(IV) domain, named Tetrastatin, exerts anti-tumor effects^19^.

We synthesized shorter peptides and demonstrated that the QS-13 peptide was still able to reproduce the effect of Tetrastatin on melanoma cells *in vitro* and *in vivo*.

Tetrastatin was shown to interact with the α_v_β_3_ integrin at the melanoma cell surface^19^. We investigated whether the QS-13 peptide was also able to interact with the α_V_β_3_ integrin. We performed cell adhesion-blocking experiments with the QS-13 peptide and demonstrated that an α_v_β_3_ blocking antibody inhibited cell adhesion. α_v_β_3_ involvement in QS-13 binding to melanoma cells was confirmed by affinity chromatography. Using β_3_ siRNA strategies, we confirmed the α_v_β_3_ integrin as a receptor of the QS-13 peptide on the SK-MEL-28 cell surface.

To further investigate the interaction between QS-13 and α_v_β_3_, *in silico* experiments were conducted. The presence of a disulphide bond in the QS-13 peptide was determined by MALDI-ToF experiments. Taken together, the results of the MD simulations emphasize the role of this disulfide bond in the structure of the different investigated peptides. The presence of the disulfide bond restrains the explored configuration space, thus leading to a lower number of clusters. In addition, the constraint imposed by the presence of the disulfide bond leads to a better exposure of the side chains (glutamine and valine central residues as well as arginine and lysine residues). When considering the interaction with α_v_β_3_, through docking experiments, we highlighted the importance of the disulfide bond since it clearly improves the values of the free energy of binding between the QS-13 peptides and integrin.

One of the hypothetical binding areas for QS-13 deduced from the docking experiments overlaps the RGD binding site^20^. The RGD peptide inhibits SK-MEL-28 cell adhesion to QS-13. These results provide evidence that the QS-13 biding site is identical or in close proximity to the RGD binding site on α_v_β_3_ and validate the first theoretical area suggested by the docking experiments.

Regarding the MD experiments, several amino-acids from the QS-13 peptide were suggested to be crucial for the interaction with α_v_β_3_. These amino-acids were sequentially replaced by an alanine residue, and modified peptides were tested for their ability to bind to the SK-MEL-28 cell surface. Substitution of the cysteines at positions 222 and 225 by alanine abolished peptide binding to SK-MEL-28. As suggested by the MD simulation and docking experiments, the disulfide bond was found to be crucial for QS-13 binding to SK-MEL-28 through α_v_β_3_. Although docking experiments were not performed with the mutated peptides, we can clearly draw a correlation between the results obtained with the MD simulations and observations extracted from the docking results. As stated previously, the MD simulations highlight the importance of the disulfide bond and its role in the structure and the exposure of the side chain groups. In particular, the right panel of Fig. 4a shows the accessibility of the arginine residue, which is a positively charged residue in a physiological environment and was simulated this way. Analysis of the docking indicates that there are two main contacts in term of integrin residues: the aspartate and glutamate residues, which are negatively charged amino acids. We can then postulate that arginine/aspartate or arginine/glutamate interactions are strong favourable electrostatic interactions that contribute to a better stabilization of the position observed in the case of QS13 peptides containing the disulfide bond since it lowers the value of the binding energy of QS13 to integrin. In summary, QS-13 binding to α_v_β_3_ appears to be conformation-dependent.

α_v_β_3_ was reported to differentially activate cell migration and the intracellular signalling FAK/PI_3_K/AKT pathway in a ligand-specific manner^24,25^. FAK (focal adhesion kinase) is a multifunctional protein^26^ that is particularly involved in tumor invasion^27^. Phosphorylation of the PI_3_K activating subunit (p85) is important, especially because of its role in cell migration and tumor growth^28^. Reiske *et al*. have shown that FAK phosphorylation at Tyr-397 promotes cell migration through PI_3_K activation^29^. PI_3_K, in turn, activates Akt. Akt is a central enzyme in tumor invasion and participates in the regulation of all stages of tumor development^30^. In our experiments, FAK^Y397^, PI_3_K p85^Y458^ and Akt^T308^ phosphorylation decreased after QS-13 binding to SK-MEL-28, in agreement with the inhibition of cell migration and invasion that we observed *in vitro*.

**Figure 7:**
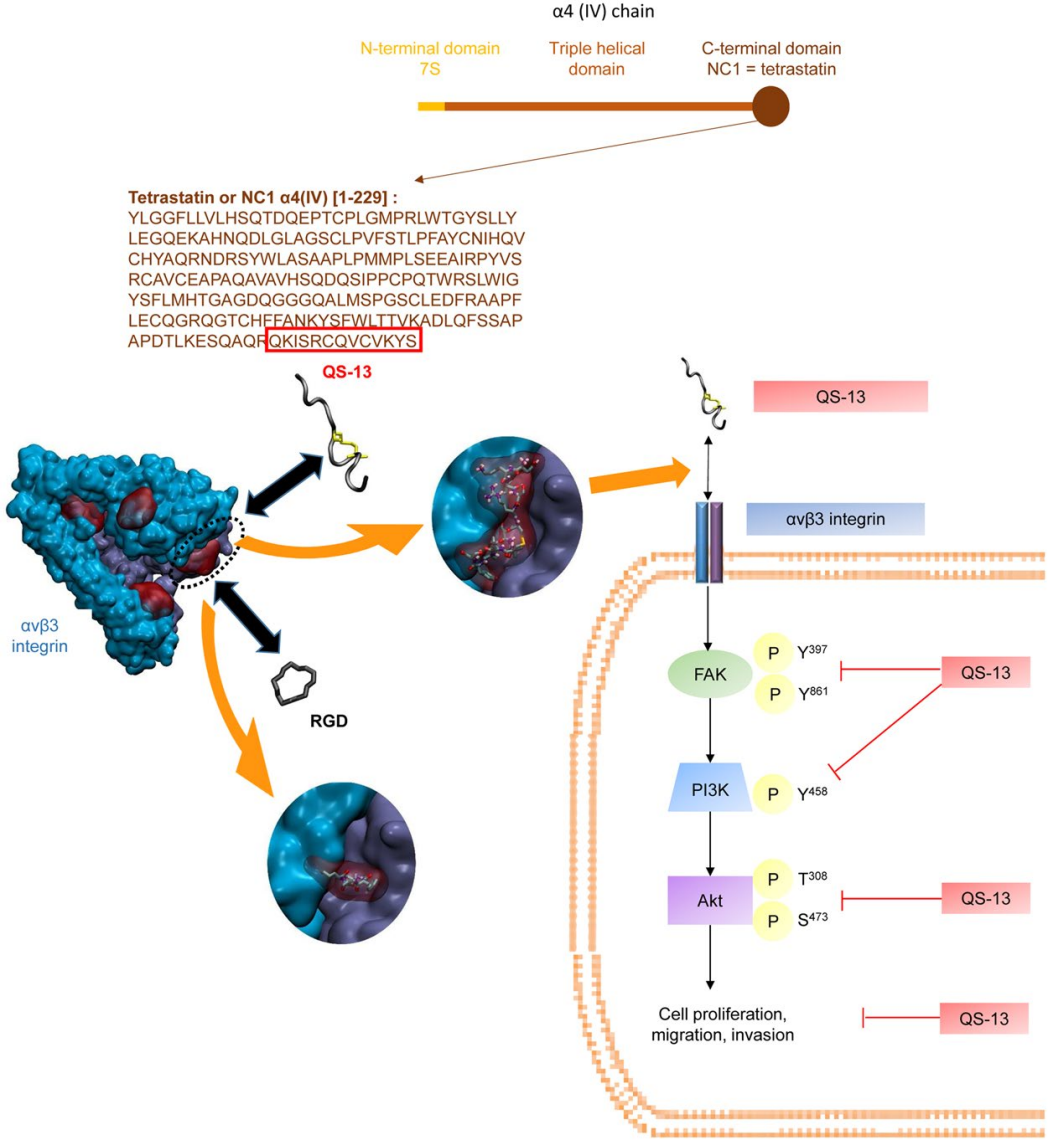
Summary diagram of QS-13 binding and mechanism of action. The QS-13 peptide is located at the C-terminal extremity of the Tetrastatin or Non Collagenous (NC)-1 domain of the α4 chain from type IV collagen. QS-13 conformation was deducted from Molecular Dynamics simulation. Molecular docking experiments suggest the presence of 5 preferred areas of interaction (PAI-1 to PAI-5); 4 of them appear in red on α_V_β_3_ integrin that is represented in blue and violet. Magnifications of PAI-1 allow to visualize QS-13 binding (up) or RGD binding (down) [20]. This explains that RGD pre-incubation abolishes QS-13 interaction with α_V_β_3_ at the cell surface. QS-13 binding inhibits cell proliferation, migration and invasion through the FAK/PI_3_K/Akt pathway.

Taken together, our results demonstrate that QS-13 strongly inhibits melanoma progression through α_v_β_3_ integrin binding (Fig. 7). This integrin is overexpressed in tumors and is a prime target for the administration of anti-cancer agents *in situ*^31^. The ultimate goal of this study is to propose a new therapeutic strategy based on QS-13 grafting on the surface of nanoparticles loaded with cytotoxic agents to deliver the drug specifically to the tumor, decreasing the required drug concentration side effects.

## Methods

### Chemicals and antibodies

Culture reagents and molecular biology products were purchased from Invitrogen (distributed by Fischer Scientific, Illkirch, France). RIPA lysis buffer, Bovine Serum Albumin, Gelatin, Matrigel and RGDS peptide were purchased from Sigma (St-Quentin, France). The anti-α_v_β_3_ integrin antibody (23C6) was purchased from Millipore (Saint Quentin-en-Yvelines, France) and the corresponding IgG1, k control isotype (MOPC-21) was from Sigma-Aldrich. Anti-phospho-FAK (Tyr397) (dilution 1/3000), anti-FAK (dilution 1/3000), anti-phospho-PI_3_K p85 (Tyr458)/p55 (Tyr199) (dilution 1/1000) antibodies were purchased from Cell Signaling (distributed by Ozyme, Saint Quentin-en-Yvelines, France). Anti-phospho-Akt (Tyr308) (dilution 1/3000) and Anti-Akt antibodies (dilution 1/3000) were purchased from Cell Signaling Technology (distributed by Ozyme, Saint Quentin en Yvelines, France). The anti-PI_3_K p85 antibody (dilution 1/2000) was purchased from Merck Millipore (Molsheim, France). The anti-actin antibody (dilution 1/10000) was purchased from Santa Cruz Biotechnology (distributed by Clinisciences, Nanterre, France). CS-50 (CHFFANKYSFWLTTVKADLQFSSAPAPDTLKESQAQRQKISRCQVCVKYS) and QS-13 (QKISRCQVCVKYS) peptides were purchased from Proteogenix (Schiltigheim, France) and used at 40 µM.

### Cell lines

SK-MEL-28 human melanoma cells and B16-F1 murine melanoma cells were obtained from ATCC. They were cultured in DMEM and 10% FBS at 37 °C in a humid atmosphere (5% CO_2_, 95% air).

### *In vivo* studies

All animal experiments were performed in level 2 animal facilities of the Faculty of Medicine and Pharmacy of Reims in accordance with the CNRS institutional guidelines (http://ethique.ipbs.fr/) and in conformity with the French Ministry of Research and Agriculture Charter on Animal Experimentation Ethics. The procedures of the animal studies were approved by the Ethics Committee in Animal Experimentation of Reims Champagne Ardenne (C2EA-56). C57Bl6 mice (6 week-old; average body weight: 18–20 g) were purchased from Harlan France (Gannat, France). Animals were individually caged and provided food and water *ad libitum*. They were housed in a room with a constant temperature and humidity. All mice were acclimatized to laboratory conditions for 1 week before starting the experiments. A suspension of B16F1 cells (2.5 × 10^5^ cells in 0.10 mL DMEM) was subcutaneously injected into the left side of C57Bl6 mice. The tumor size was measured at days 10, 15, and 20. Mice were sacrificed at day 20 and the tumor sizes were measured. Each group contained at least 7 mice. The tumor volumes were determined according to v = ½ A × B^2^, where A denotes the largest dimension of the tumor and B represents the smallest dimension^32^.

### Expression and Purification of Recombinant Human Tetrastatin

Recombinant human Tetrastatin was prepared as described in a previous paper^19^.

### *In vitro* proliferation assays

SK-MEL-28 proliferation was evaluated using the WST-1 reagent as described in a previous paper^19^.

### Anchorage-independent growth

Soft agar assays were carried out as described in a previous paper^19^.

### Scratch wound assay

SK-MEL-28 cells (5 × 10^4^ per well) were seeded in 24-well plates and grown to confluence in DMEM supplemented with 10% FBS. The cell layer was then wounded with a sterile 100 to 1000 μL pipet tip and re-incubated in fresh culture medium in the absence or presence of peptide. After 72 h, cells were photographed and the size of the remaining wound was measured.

### *In vitro* invasion assays

Invasion was assayed in modified Boyden chambers (tissue culture treated, 6.5 mm diameter, 8 μm pore, Greiner-One, Courtaboeuf, France) as described in a previous paper^19^.

### Adhesion assays

Cells were detached with 50 mM Hepes, 125 mM NaCl, 5 mM KCl and 1 mM EDTA, washed three times with DMEM and preincubated for 30 min with different effectors. 2 × 10^4^ cells were seeded per well on a 96 well-plate that was previously coated with QS-13 peptide. After 60 min of adhesion, cells were washed three times with PBS, fixed with 1.1% glutaraldehyde and stained with crystal violet. After elution with 10% acetic acid, absorbance was read at 560 nm.

### Flow cytometry analysis

Cells were detached with 50 mM Hepes, 125 mM NaCl, 5 mM KCl and 1 mM EDTA and washed three times with DMEM. They were incubated for 30 min with different biotinylated peptides and washed three times with DMEM. They were incubated for 30 min with Alexa Fluor 488 Streptavidin and washed three times with DMEM. They were analysed by a FACS Fortessa flow cytometer (BD Biosciences, San Jose, USA). Ten thousand cells, gated for forward scatter vs. side scatter, were collected for each sample. The results were analysed using Flow Jo software (FlowJo Enterprise, Ashland, OR, USA). Quantification of fluorescence was performed using the median fluorescence intensity (MFI).

### Affinity chromatography analysis

SK-MEL-28 protein extracts were chromatographed at 4 °C on a HiTrap NHS-activated Sepharose High Performance column (GE Healthcare™, Orsay, France) that was previously functionalized with the QS-13 peptide according to the manufacturer instructions. Unbound material was removed by washing with 30 mL of washing buffer (10 mM Tris, 1 mM CaCl_2_, 1 mM MgCl_2_, pH 7.6, 1 mM N-Ethylmaleimide (NEM), 1 mM phenylmethylsulfonyl fluoride (PMSF) and 0.1% octylglucoside). Proteins bound to the affinity column were then eluted with elution buffer: 10 mM Tris, pH 7.6, 1/100 PIC (ProteoBlock Protease Inhibitor Cocktail, Fermentas™, Illkirch, France) (w/v) and 0.1% octylglucoside supplemented with increasing concentrations of NaCl (0.15, 0.6 and 1 M). Eluted samples were then solubilized in SDS sample buffer with 10 mM DTT, denatured at 95 °C for 5 min and submitted to SDS-PAGE.

### Western blot analysis

Cells were incubated for 10 min with RIPA lysis buffer (1% (v/v) Triton X-100, 1% (v/v) sodium deoxycholate, 0.1% (v/v) SDS, 20 mM Tris–HCl, pH 7.4, 150 mM NaCl, and 1 mM EDTA in distilled water supplemented with the Halt™ Protease and Phosphatase Inhibitor Cocktail (Thermo Fisher Scientific, Illkirch, France) and scraped. Samples were mechanically resuspended by pipetting and vortexing every 5 min for 30 min. After that, they were centrifuged at 10 000 g for 10 min at 4 °C, and the resulting supernatants were quantified using a Bradford assay. Fifty micrograms of each sample was diluted in sample buffer, reduced using β-mercaptoethanol and heated at 95 °C for 10 min. Samples were analysed by western blotting as described in a previous paper^19^.

### RT-qPCR

Total RNA isolation was performed using a Qiagen RNeasy kit (Qiagen, Courtaboeuf, France) according to the manufacturer’s instructions. cDNA were prepared from 100 ng of total RNA by reverse transcription (RT) using a Maxima First Strand cDNA Synthesis Kit (Thermo Fisher Scientific, Villebon-sur-Yvette, France) according to the manufacturer’s protocol. Real-time PCR was performed using a Maxima SYBR green/rox qPCR master mix (Thermo Fisher Scientific, Villebon-sur-Yvette, France) on the Stratagene Mx3005P qPCR detection system (Agilent technologies, Les Ulis, France). The PCR conditions were 10 min at 95 °C, followed by 40 cycles each consisting of 15 s at 95 °C (denaturation), 30 s at 60 °C (annealing) and 30 s at 72 °C (extension). After real-time PCR, melting curve analysis was performed by continuously measuring fluorescence during heating from 55 to 95 °C at a transition rate of +0.2 °C/s. Product specificity was evaluated by melting curve analysis and electrophoresis on 2% agarose gels. Fluorescence was analysed using the Data Analysis software (Stratagene). Crossing points (Cp or Ct) were established using the second derivative method. The real-time PCR efficiency was calculated from the slope of the standard curve. The target gene expression levels were normalized to the reference gene.

The following primers were used: ITGB3 forward primer: 5′-gacaagggctctggagacag-3′ and reverse primer: 5′-actggtgagctttcgcatct-3′; eEF1A1 forward primer: 5′-ctggagccaagtgctaacatgcc-3′ and reverse primer 5′-ccgggtttgagaacaccagtc-3′.

### siRNA transfection

Human beta 3 integrin subunit-specific siRNA and negative control siRNA (non-targeting pool) (FlexiTube GeneSolution) were purchased from Qiagen (Courtaboeuf, France). The siRNA targeted different regions of the ITGB3 mRNA: the 1st siRNA target sequence (5′-cacgtgtggcctgttcttcta-3′), 2nd siRNA target sequence (5′-caagctgaacctaatagccat-3′), 3rd siRNA target sequence (5′-ctctcctgatgtagcacttaa-3′) and 4th siRNA target sequence (5′-ccgcttcaatgaggaagtgaa-3′). Exponentially growing SK-MEL-28 cells were transfected with siRNA pools (20 nM) using lipofectamine™ 2000. The decrease in ITGB3 mRNA expression was measured by RT-qPCR 24 h after transfection. The decrease in β_3_ integrin protein expression was assessed 48 h after siRNA transfection. Adhesion assays on the QS-13 peptide were performed 48 h after siRNA transfection.

### Matrix assisted laser desorption ionisation – Time of flight (MALDI – ToF)

Solutions of matrix α–cyano-4-hydroxycinnamic acid (CHCA) were prepared as a saturated solution in ACN/water (1/1; v/v) with 0.1% TFA. Samples were prepared at a concentration of 10 pmol/μL in water containing 0.1% TFA. Typically, 1 μL of the matrix was pipetted onto the MALDI target plate; then 1 μL of analyte was added and air-dried for MALDI-ToF MS analysis. MS experiments were performed using a micromass MALDI^TM^–LR Time-of-Flight mass spectrometer (Waters MS technologies, Manchester, UK) equipped with a nitrogen UV laser (337 nm wavelength), reflectron optics, fast dual micro-channel plate (MCP) detector and high magnification camera system. Positive ion spectra were acquired in reflectron mode. The following voltages were applied: pulse 1940 V, MCP 2380 V, suppression 500, flight tube 12000 V, and reflectron 5200 V. A time lag focusing (TLF) delay of 680 ns was used between the time of laser pulse and application of the accelerating voltage. Samples were analysed in reflectron acquisition mode in the mass range from 550–4000 Da. Mass calibration was performed using a peptide mixture composed of Bradykinin fragment 1–5 (573.31 Da), human Angiotensin II (1046.54 Da), Neurotensin (1672.92 Da), ACTH clip (2465.20 Da), bovine Insulin ß-chain oxidized (3494.65 Da) and bovine Insulin (5730.61 Da).

### Molecular dynamics simulations

Molecular dynamic simulations on the different peptides were conducted using the GROMACS simulation package^33,34^. The OPLSAA force field was chosen as the set of parameters for describing the atoms and their interactions^35,36^. Isolated peptides were placed in boxes with side lengths varying from 40 Å to 190 Å depending on their size. These values were chosen so that a given peptide would not interact with its images when the periodic boundary conditions were applied. Water (TIP3P model^37^) and Cl^−^ counter ions were added prior to the simulations. To relax the structures, 5000 steps of energy minimization were performed using the steepest descent algorithm. The systems were equilibrated for 500 ps at a temperature of 310 K in the isothermal-isobaric ensemble. The equilibration steps were followed by molecular dynamics (MD) simulations carried out for 200 ns, maintaining a pressure of 1 bar (Berendsen algorithm) and temperature of 310 K (V-rescale algorithm). The Verlet algorithm was used to integrate the equation from classical mechanics in parallel with an integration step of 2 fs since the length of the bonds implicating hydrogen atoms were frozen using the SHAKE algorithm^38^. For computation of the non-bonded interactions, the Particle Mesh Ewald (PME) algorithm^39,40^ was used with a cut off at 1.8 Å for the coulombic interactions and a potential-shifting function for van der Waals interactions applied at 1.3 Å and a cut off at 1.4 Å.

The trajectories of the QS-13 peptides were analysed using the clustering tool available in the GROMACS simulation package. Each of the trajectories was decomposed using the g cluster module of the GROMACS simulation package and the gromos algorithm. In order to form the different clusters or families of structures, RMSD were calculated based on the positions of the α-carbons of the peptides and a cutoff of 3 Å was set. This cutoff was chosen because it gave a good compromise between the total number of clusters obtained and also the distributions of the structures within these clusters.

### Docking experiments

Docking of the QS-13 peptides against the α_v_β_3_ integrin (RCSB Protein Data Bank 4G1M) was performed using Autodock software (version 4.2)^41^. We extracted the structure of the RGD peptide (which is known to bind to integrin α_v_β_3_) from the PDB structure 1L5G and performed preliminary docking experiments to determine the relevant set of docking parameters. The software was used with a fixed integrin and semi-flexible QS13 ligand (the backbone was frozen as well as the amide links and guanadinium groups). Since the integrin is a large molecule, we performed several independent dockings targeting different subvolumes of the protein; we considered 180 overlapping boxes with a volume of 47.25 Å × 47.25 Å ×  47.25 Å. Each box was divided along the three directions, and the distance between the nodes was equal to 0.375 Å. The Lamarckian genetic algorithm was used, and for each ligand, 150 dockings were performed with the default parameters of Autodock except for the population size (150), number of energy evaluations (5 × 10^6^), and maximum number of generations (30, 000), which were derived from the preliminary study. Molecular models were graphed with VMD software, which is available online.

### Quantification and statistical analyses

For *in vitro* experiments, results are expressed as the mean ± SD and were analysed using Student’s *t*-test. For *in vivo* experiments, the volumes of the primary tumors were analysed using the non-parametric Kruskal-Wallis test.

## Electronic supplementary material

Supplementary Information
