# Supplementary material for: Conformation-dependent binding of a Tetrastatin peptide to αvβ3 integrin decreases melanoma progression through FAK/PI3K/Akt pathway inhibition
Source: Sci Rep. 2018 Jun 29;8:9837. doi: 10.1038/s41598-018-28003-x (PMC6026150; doi:10.1038/s41598-018-28003-x)
Supplement: Supplementary file 1 — Supplementary Information [file 41598_2018_28003_MOESM1_ESM.pdf]

TITLE

Conformation-dependent binding of a Tetrastatin peptide to $\alpha_v\beta_3$ integrin decreases melanoma progression through FAK/PI$_3$K/Akt pathway inhibition.

AUTHORS/AFFILIATIONS

Eléonore Lambert[1,5], Eloïse Fuselier[1], Laurent Ramont[1,2], Bertrand Brassart[1], Sylvain Dukic[1], Jean-Baptiste Oudart[1,2], Aurélie Dupont-Deshorgue[1], Christèle Sellier[1], Carine Machado[3], Manuel Dauchez[1,4], Jean-Claude Monboisse[1,2], François-Xavier Maquart[1,2], Stéphanie Baud[1,4], Sylvie Brassart-Pasco[*,1].

[1]UMR CNRS/URCA 7369, Matrice Extracellulaire et Dynamique Cellulaire (MEDyC), Université de Reims Champagne Ardenne (URCA), Reims, F-51100, France.

[2]CHU de Reims, Laboratoire Central de Biochimie, Reims, F-51092, France

[3]CNRS UMR 7312, Institut de Chimie Moléculaire de Reims, Université de Reims Champagne Ardenne (URCA), Reims, F-51100, France.

[4]Plateau de Modélisation Moléculaire Multi-échelle, Université de Reims Champagne Ardenne (URCA), Reims, F-51687, France.

[5]Present address: Laboratoire de Recherche sur les Nanosciences (LRN), EA4682, Université de Reims Champagne-Ardenne, Reims, F-51100 Reims, France.

*Corresponding Author**:** Dr. S. BRASSART-PASCO, Laboratoire de Biochimie Médicale et de Biologie Moléculaire CNRS UMR 7369, U.F.R. Médecine, 51 Rue Cognacq Jay, 51095 REIMS Cedex, France. Phone: 33.3.26.91.35.32; Fax: 33.3.26.91.80.55; Email: sylvie.brassart-pasco@univ-reims.fr.

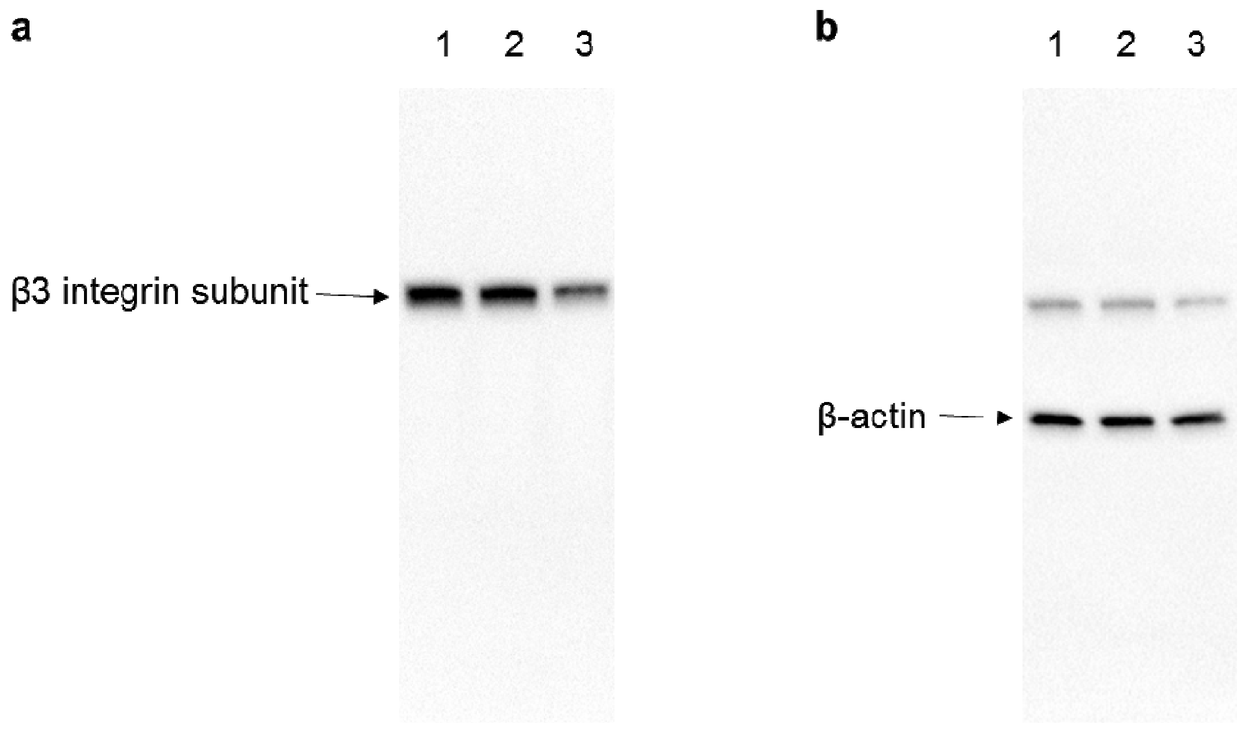

Fig. S1: SiRNA targeting of β_3 integrin subunit.
Cells were transfected with β_3 subunit siRNA or control siRNA. Western blot were performed on cell extracts 48 h after transfection using anti-β_3 integrin antibody (a) or anti-β-actin antibody. Lane 1: control; lane 2: control siRNA; lane 3: β_3 subunit siRNA.

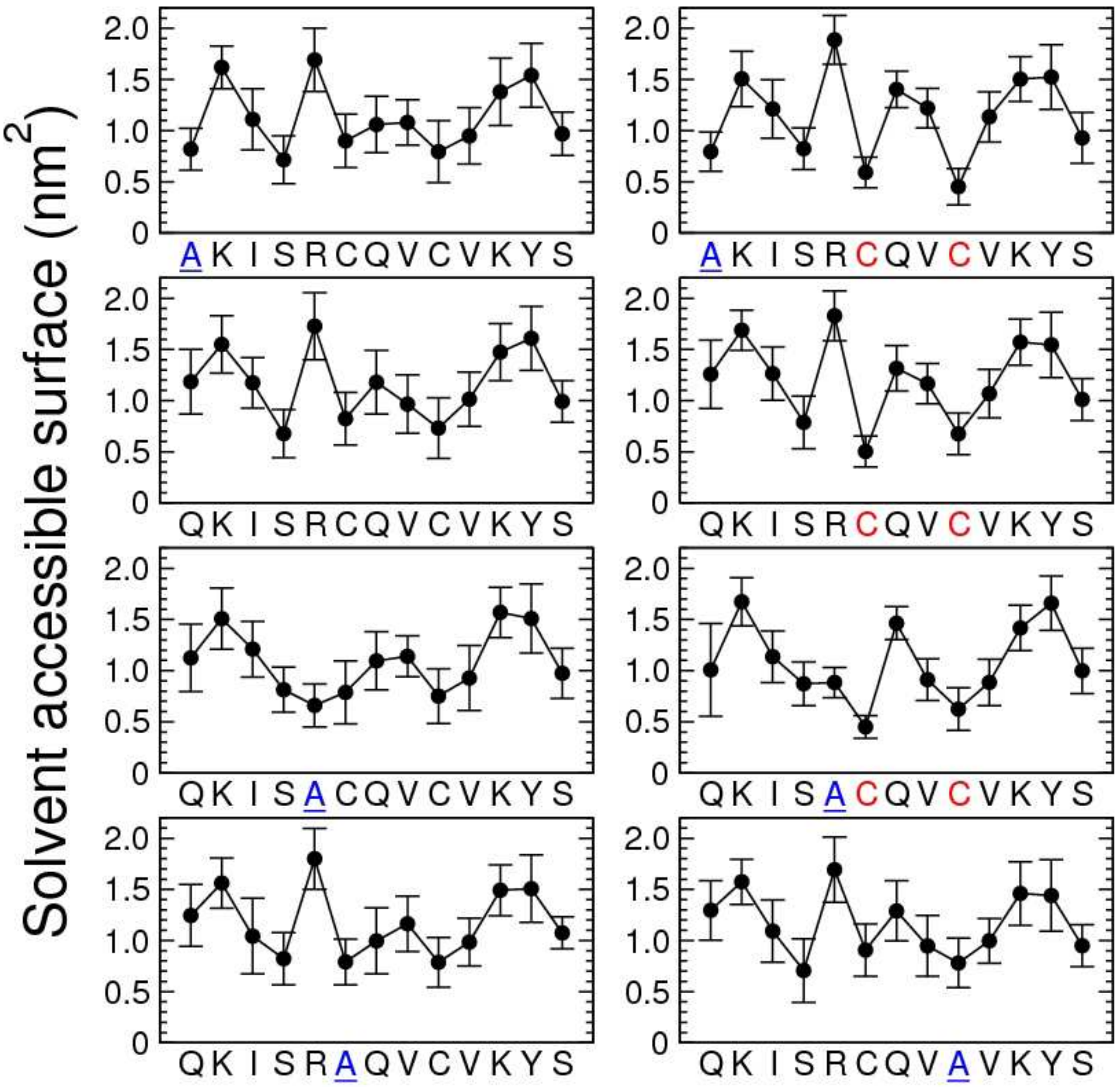

Fig. S2: Solvent accessible surface (in nm^2) computed as a function of the sequence.
From left to right and top to bottom, the following peptides are considered: AS-13, AS-13-db, QS-13, QS-13-db, QS-13-R5A, QS-13-R5A-db, QS13-C6A, QS-13-C9A. Mutated residues are displayed in underlined blue letters and the Cysteine residues forming a disulfide bond are highlighted in red.

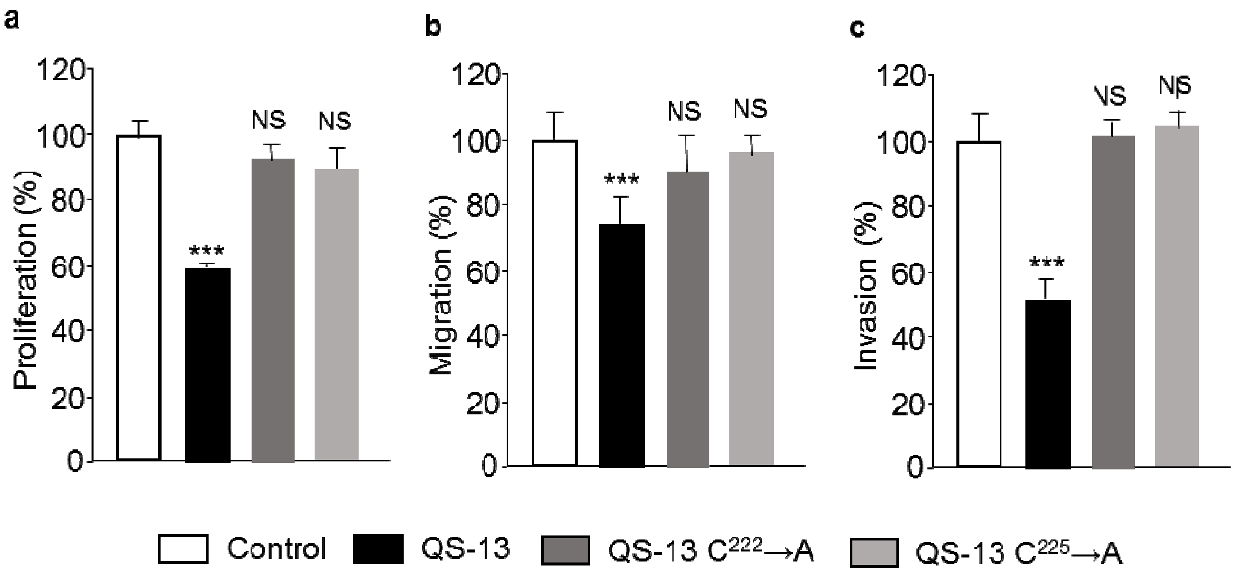

Fig. S3: Substitution of C^{222} or C^{225} abolishes QS-13 anti-tumor *in vitro* effects.
Cell proliferation was measured after 72 h of incubation **(a)**. Cell migration in scratch wound assay was measured after 48 h of incubation. **(b)**. Cell invasion through Matrigel-coated membranes was measured after 48 h of incubation. **(c)**. ***: $p<0.001$.

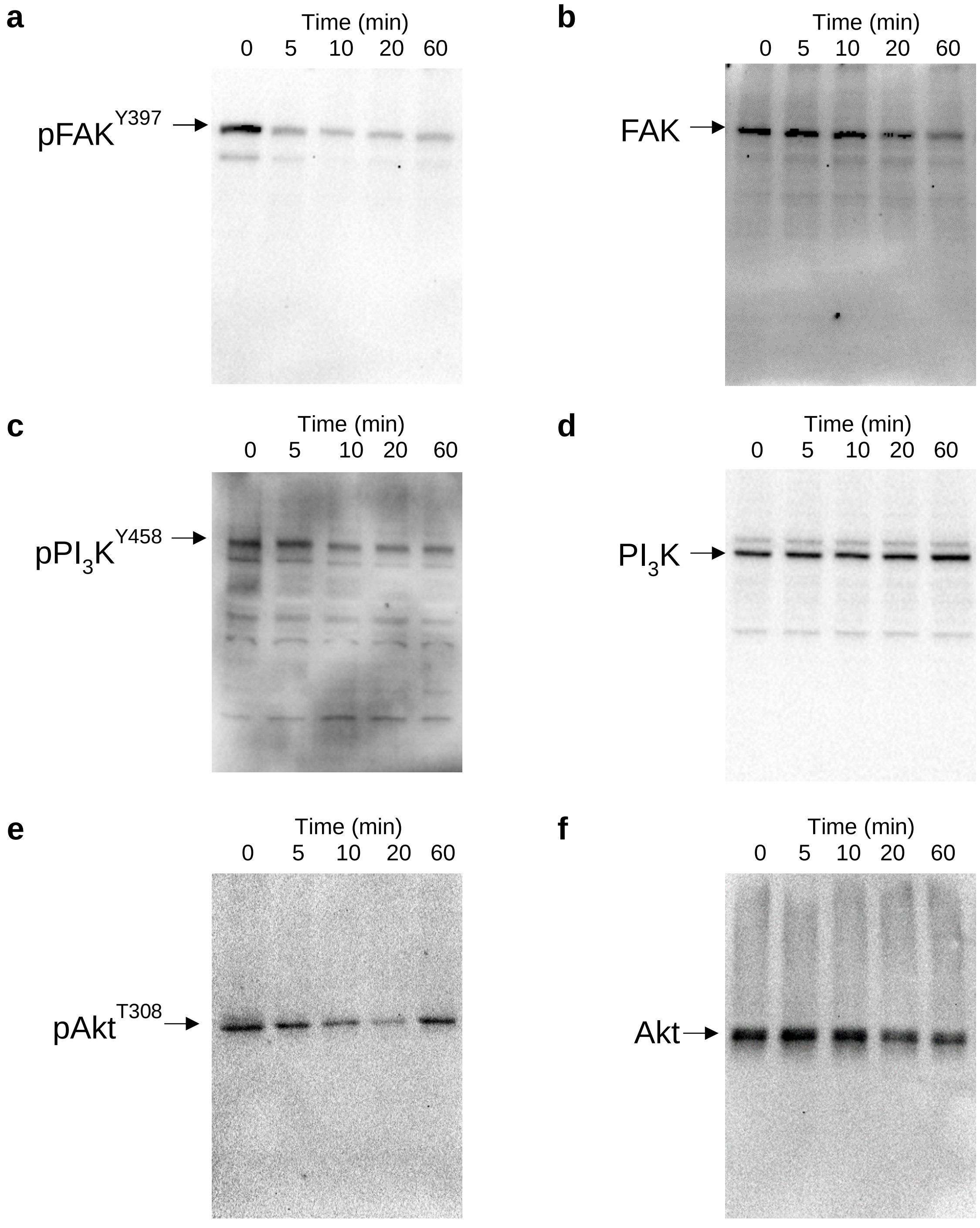

Fig. S4: Transduction pathway analysis.
Western blot analysis of phosphorylated-FAK^{Y397} (a), total FAK (b), phosphorylated-PI$_3$K p85^{Y458} (c) total PI$_3$K p85 subunit (d), phosphorylated-Akt^{T308} (e), total Akt (f).

Peptide	Description	Sequence
QS-13-db	C-terminal 13 amino-acid sequence of Tetrastatin with a disulfide bond between ^{222}C and ^{225}C	QKISR**C**QV**C**VKYS
QS-13	C-terminal 13 amino-acid sequence of Tetrastatin without disulfide bond	QKISRCQVCVKYS
AS-13-db	Mutated C-terminal 13 amino-acid sequence of Tetrastatin with a disulfide bond between ^{222}C and ^{225}C	**A**KISR**C**QV**C**VKYS
AS-13	Mutated C-terminal 13 amino-acid sequence of Tetrastatin without disulfide bond	**A**KISRCQVCVKYS
QS-13-AR5-db	Mutated C-terminal 13 amino-acid sequence of Tetrastatin with a disulfide bond between ^{222}C and ^{225}C	QKIS**AC**QV**C**VKYS
QS-13-AR5	Mutated C-terminal 13 amino-acid sequence of Tetrastatin without disulfide bond	QKIS**A**CQVCVKYS
QS-13-C6A	Mutated C-terminal 13 amino-acid sequence of Tetrastatin without disulfide bond	QKISR**A**QVCVKYS
QS-13-C9A	Mutated C-terminal 13 amino-acid sequence of Tetrastatin without disulfide bond	QKISRCQV**A**VKYS

Table S1: Peptides investigated through MD simulations.
The presence of the disulfide bond is highlighted with the underlined Cysteine residues (C). Mutations performed on the QS13 peptides are represented in bold characters.

Peptide	**Number of clusters**	**Percentage of structures in the five first clusters**
QS-13-db	33	75.3
QS-13	61	58.8
AS-13-db	36	73.1
AS-13	53	58.2
QS-13-R5A-db	26	89.5
QS-13-R5A	57	69.3
QS-13-C6A	57	66.4
QS-13-C9A	51	73.7

Table S2: Results of the clustering experiments.
For each MD simulation, the total number of clusters as well as the percentage of structures found in the five first clusters are given.

**Peptide**	**PAI-1**	**PAI-2**	**PAI-3**	**PAI-4**	**PAI-5**
QS-13-1	13.9 (**-0.60**)	27.8 (**-0.23**)	6.1 (**-0.65**)	15.0 (**-1.63**)	28.9 (**-4.31**)
QS-13-2	16.1 (**-3.74**)	16.1 (**-2.39**)	0.0	29.4 (**-1.53**)	25.6 (**-2.79**)
QS-13-3	20.6 (**-1.22**)	23.3 (**-0.70)**	4.4 (**0.32**)	12.8 (**-0.66**)	30.6 (**-2.14**)
QS-13-4	5.6 (**-1.24**)	11.7 (**-0.35**)	10.6 (**-0.52**)	20.6 (**-0.19**)	36.7 (**-1.14**)
QS-13-5	10.0 (**-3.16**)	14.4 (**-2.86**)	17.8 (**-4.45**)	15.0 (**-4.32**)	31.7 (**-4.20**)
QS-13-6	8.3 (**-3.72**)	23.9 (**-6.89**)	18.3 (**-5.93**)	3.3 (**-3.33**)	16.7 (**-4.60**)
**Average**	12.4 (**-3.74**)	19.5 (**-6.89**)	9.5 (**-5.93**)	16.0 (**-4.32**)	28.4 (**-4.60**)

Table S3: Characterization of the PAIs.
For each peptide, the frequency of appearance in a given PAI after the docking experiment was evaluated as well as the lowest free energy of binding (in parenthesis) associated to the PAI.
